# Effects of temperature and water turbulence on vertebral number and body shape in *Astyanax mexicanus* (Teleostei: Characidae)

**DOI:** 10.1371/journal.pone.0219677

**Published:** 2019-07-29

**Authors:** Winer Daniel Reyes Corral, Windsor E. Aguirre

**Affiliations:** Department of Biological Sciences, DePaul University, Chicago, Illinois, United States of America; Laboratoire de Biologie du Développement de Villefranche-sur-Mer, FRANCE

## Abstract

Environmental changes can modify the phenotypic characteristics of populations, which in turn can influence their evolutionary trajectories. In ectotherms like fishes, temperature is a particularly important environmental variable that is known to have significant impacts on the phenotype. Here, we raised specimens of the surface ecomorph of *Astyanax mexicanus* at temperatures of 20°C, 23°C, 25°C, and 28°C to examine how temperature influenced vertebral number and body shape. To increase biological realism, specimens were also subjected to two water turbulence regimes. Vertebral number was counted from x-rays and body shape variation was analysed using geometric morphometric methods. Temperature significantly impacted mean total vertebral number, which increased at the lowest and highest temperatures. Fish reared at lower temperatures had relatively more precaudal vertebrae while fish reared at higher temperatures had relatively more caudal vertebrae. Vertebral anomalies, especially vertebral fusions, were most frequent at the extreme temperature treatments. Temperature significantly impacted body shape as well, with fish reared at 20°C being particularly divergent. Water turbulence also impacted body shape in a generally predictable manner, with specimens reared in high turbulence environments being more streamlined and having extended dorsal and anal fin bases. Variation in environmental variables thus resulted in significant changes in morphological traits known to impact fish fitness, indicating that *A*. *mexicanus* has the capacity to exhibit a range of phenotypic plasticity when challenged by environmental change. Understanding the biochemical mechanisms underlying this plasticity and whether adaptive plasticity has influenced the evolutionary radiation of the Characidae, are major directions for future research.

## Introduction

Phenotypic plasticity describes the ability of a genotype to produce a range of phenotypes in response to interactions with the environment. Phenotypic plasticity is often the first form of response to changes in the environment and may play an important role during the early stages of adaptation to new habitats [1–7]. Phenotypic plasticity itself may also evolve when there is heritable variation among individuals in the propensity or actual nature of the plastic response [8]. In addition, favourable plastic traits may eventually become integrated into the genetic machinery of populations that colonize novel environments [9,10]. Thus, studying phenotypic plasticity is important for understanding how phenotypic variation is generated and may also provide insight into the evolutionary potential of lineages [7,11].

As ectotherms, bony fishes are particularly susceptible to variation in environmental conditions during development, especially temperature. Environmental conditions can change quickly in some freshwater ecosystems, leading to strong variation of environmental conditions over small geographic distances. Combined with other factors like reduced rates of gene flow, this environmental heterogeneity has contributed to the high rates of fish diversity in freshwater ecosystems throughout the world [12]. For example, in Neotropical streams with great fish diversity, environmental variables like nutrient concentration, water velocity, and water temperature can vary considerably along elevational gradients [13]. With these changing environmental conditions come changes in the composition of the fish communities [13,14] and even the phenotypic properties of species occurring over broad elevational ranges [15]. Water temperature is a particularly important factor influencing the diversity and phenotypic properties of fishes and one of the most commonly studied environmental factors in relation to phenotypic plasticity. Temperature during development can impact an enormous array of phenotypic and physiological characteristics in fishes, including vertebral number and body shape, which can have important consequences for fitness [16–24]. Vertebral number and body shape are associated in fishes–both within and between species–such that fish with more elongate bodies tend to have more vertebrae [25–31].

Knowledge that change in temperature during development impacts vertebral number dates back to the late 1800s when Jordan [32] documented an association between increasing latitude and vertebral number (Jordan´s rule). Although the actual relationship varies among species, variation in temperature during development generally results in an inverse relationship between vertebral number and temperature or in a U-shaped pattern, with vertebral number being greater at temperature extremes and lower at intermediate temperatures [33–35]. Beyond impacting total vertebral number, temperature may also impact the proportion of precaudal to caudal vertebrae, which varies widely in fishes [36] and is associated with adaptively important traits like predator escape ability [37] and predation mode [38]. In addition, there have been reports of decreased body depth in fishes grown at lower developmental temperatures [39], which indicates that variation in temperature may sometimes result in covariation between adaptively important traits like vertebral number and body shape (more vertebrae and more streamlined bodies at lower temperatures and vice-versa).

The present study seeks to examine the relationship between water temperature, turbulence, vertebral number and body shape in the surface ecomorph of the emerging model species, *Astyanax mexicanus* [40–42]. *Astyanax mexicanus* includes two highly divergent ecomorphs, a surface and a cave ecomorph corresponding to distinct populations that have diverged in response to extreme differences in habitat conditions between surface and cave rivers. Because of the magnitude of morphological, physiological and ecological differences between the ecomorphs, *A*. *mexicanus* has attracted great attention from developmental and evolutionary biologists.

We also tested for the effects of water turbulence because it is another important environmental factor that can influence the phenotypic characteristics of fishes and varies widely across ecosystems. Temperature and water turbulence can also covary along elevational gradients in streams. At higher elevations, streams are often colder and exhibit greater water velocity while at lower elevations, they tend to be warmer and run more slowly as they broaden [43,44]. Body shape is a trait that is particularly susceptible to variation in water turbulence because of the energetic demands associated with swimming in a highly viscous aquatic environment [45–47]. Previous studies have demonstrated the effects of water turbulence on body shape across taxonomically diverse groups, with fish exposed to high water turbulence rates tending to be more streamlined in order to reduce the drag caused by the current [23,48–50].

The main hypotheses tested in this study are that temperature differences during early development will affect vertebral phenotypes and that differences in water turbulence following the temperature treatments will result in divergence in body shape in an adaptive direction: fish in the added turbulence treatment will be more streamlined. A negative or a U-shaped relationship between developmental temperature and vertebral number is predicted, as seen in other fish species. Temperature may additionally result in changes in the ratio of precaudal to caudal vertebrae and body shape. However, if present, we expect the effect of temperature on body shape will be less pronounced than on vertebral number because there are numerous studies documenting how temperature impacts vertebral number in fishes [24,51–56] and relatively few studies that report temperature effects on body shape [21,57–59]. We also expect that fish reared in the turbulent water treatment will develop more streamlined bodies, but we do not expect the turbulence treatment to significantly affect vertebral number because vertebral number is set very early in development by the number of somites that form, which occurs by the time eggs hatch, well before the turbulence treatment was applied in this experiment [60,61]. Finally, if vertebral number is significantly associated with body shape variation, we predict that fish with low vertebral counts will be deeper bodied while fish with high vertebral counts will be more streamlined.

## Materials and methods

### Fish maintenance and breeding

All procedures were approved by Institutional Animal Care and Use Committee (IACUC), of DePaul University (DePaul IACUC Protocol#2014–001). One-year old individuals of the surface ecomorph of *A*. *mexicanus* were obtained from the Jeffery Laboratory at the University of Maryland. The individuals used were part of a line established in 2002 (4^th^ generation) from wild-caught specimens collected in Rio Grande, Texas National Park. Individuals were separated by sex (12 males and 12 females) and kept in two 75.5 litres tanks filled with treated tap water (pH 7, 800μS conductivity). Adults were fed with commercial tetra flakes (Tetramin) and maintained on a 14-hour light/10-hour dark light cycle. Before breeding, females were supplemented with a high fat content diet using egg yolk flakes (Pentair) for 14 days, following Borowsky [62] (males did not need to be conditioned for breeding).

To obtain embryos for the experiment, on the morning of August 21, 2014 we separated breeding pairs into 20.8 litres tanks with clean water, at a stable temperature of 21°C. To induce breeding by natural spawning, the temperature was raised three degrees (24°C) [62]. Tanks were checked continuously for eggs after the room was dark and spawning occurred around midnight in seven out of ten intended crosses, each involving different pairs of adults.

### Environmental treatments setup

As soon as eggs were spawned, we removed the parents and collected the eggs from the bottom of the tanks using a siphon. The eggs from the crosses were pooled and then evenly distributed (approximately 200 per treatment) into four temperature treatments of 20°C, 23°C, 25°C, and 28°C (all ±1°C), where 23°C and 25°C are considered average for the species [63]. The 23–28°C temperature treatments were maintained using 25-watt aquarium heaters (Marineland Stealth: ETP25-25W) on a 12h light/dark cycle. For the 20°C temperature treatment, eggs were incubated in a VWR Scientific Model 2015 incubator on a 12h light/dark cycle. Each tank had an adhesive thermometer and temperature was checked daily while feeding using a digital thermometer. To facilitate egg monitoring while developing, we kept groups of eggs in two petri dishes per tank per treatment until they hatched, which varied between approximately 24 hours post-fertilization for the 28°C treatment to approximately 48h for the 20°C treatment. Infertile and dead eggs were removed following Borowsky [62]. Once they hatched, the fry was distributed into eight 20.8 litres tanks per temperature treatment for a total of 32 tanks used to rear the fry. Each tank was aerated using Maxima A-805 air pumps attached to a Pentair HF1 HydroSponge filter. Larvae were fed commercial brine shrimp nauplii once they lost their yolk sac (approximately 5 days post-fertilization depending on temperature) until they reached approximately 12 mm in total length (approximately 2 weeks post-fertilization depending on temperature). They were then gradually fed more finely ground versions of an adult diet until they began to take full flake food (4 weeks post-fertilization). Temperature treatment was continued for 4 weeks to ensure that both somite and vertebral number were completely set. Somite number is established by when eggs hatch, which typically happens within 24–48 hours of fertilization at these temperatures in *A*. *mexicanus* and vertebral number is a function of the number of somites [64]. The temperature treatment was discontinued after four weeks because the primary goal of the experiment was to examine the effect of temperature differences during early development, when vertebral number is established, on the phenotype.

After four weeks in the temperature treatments, all fish were kept at 23°C and the water turbulence treatment began. The water turbulence treatment consisted of adding a submersible water pump (VicTsing 80 GPH) attached to one end of the tank to induce water movement and was applied to half the tanks (n = 4) of the former temperature treatment groups. As occurs in natural settings, water flow was variable in the tanks. Maximum water speed in front of the pump was approximately 0.26 m s^-1^, while other speeds measured at different depths away from the sides of the tanks ranged from 0.16–0.21 m s^-1^ (See S1 Appendix for details on the calculations). Water velocities were likely much lower along the sides of the tanks and water in the corners of the tank may have been functionally equivalent to still water. The treatment was maintained for 16 weeks, when fish were large enough to x-ray, all morphological features were developed enough to facilitate data collection, and body shape was similar to that of adults. Throughout the paper, the no turbulence and water turbulence treatments refer to this extra turbulence treatment resulting from the submersible water pumps. All tanks were aeriated with air pumps attached to hydrofilters and thus had some water movement in them, but we use the terms turbulence and no turbulence for brevity. Partial (33%) water changes were conducted at least once a week and typically twice a week to maintain high water quality. Water chemistry (ammonia, nitrates, nitrites, etc.) was monitored weekly and health of all individuals was monitored daily by the animal facility staff and researchers. Research staff was trained to handle the fish, provide routine care, and identify sick and distressed animals.

### Data collection

Individuals were removed manually with a fish net from the treatments as they grew based on the density per tank to prevent severe competition for food and stimulate growth. Transfer events occurred twice, first in October and then in November of 2014. The experiment lasted from August 21, 2014 to December 21, 2014 (20 weeks). The goal was to leave ten specimens per tank for data collection. The transferred individuals were moved to other tanks to maintain the colony or euthanized using a solution of 500mg/L of buffered tricaine methanesulfonate (MS-222, pH 7.5) but were not included in the experiment.

Experimental tanks achieved a final density of 10 fish per tank except in tanks in which fish died of natural causes after the final transfer event. Most tanks (n = 27) had ten specimens in them at the end of the experiment, four had nine specimens, and only one had seven specimens in it. Of these five tanks with less specimens, three were from the 20°C treatment (two of which were turbulence treatments and the other was in the no turbulence treatment) and two were from the 28°C treatment (one each from the turbulence and no turbulence treatments). The average size of fish in these tanks with fewer specimens was significantly larger than those with a final sample size of 10 specimens (t-test, t = 2.77, df = 30, P<0.01) with the difference being about 3 mm in standard length, so body size was used as a covariate in the analysis of the body shape data. Vertebral number is set very early in development, so it is not sensitive to the differences in post-hatching growth rate seen here.

After the 20-week period, a total of 313 individuals that were included in the experimental treatments were euthanized using a solution of 500mg/L of buffered tricaine methanesulfonate (MS-222, pH 7.5). Fish were left in the solution for ten minutes after opercular movement ceased, then transferred to 10% formaldehyde for fixation (24 hours minimum), and finally preserved in 70% ethanol. The euthanizing process was done in one day. The average size of the specimens analysed was 32.3 mm standard length (SL: measured from the tip of the snout to the base of the caudal fin), with tank averages ranging from 27.6 to 38.8 mm SL. A factorial ANOVA was performed in VassarStats (http://vassarstats.net/) to examine whether tank mean SL differed among treatments. Tank mean SL did not differ between turbulence treatments (F = 0.77, df = 1, 24, P = 0.389) or the interaction between temperature and turbulence treatments (F = 1.01, df = 3, 24, P = 0.406), but did differ among temperature treatments (F = 5.00, df = 3, 24, P = 0.008). Fish in the 20°C treatment (mean = 34.64+0.75 mm SL (mean of eight tanks + standard error of the mean)) were significantly larger than those in the 23°C (mean = 30.97+1.10 mm SL, P<0.05) and 28°C (mean = 31.20+1.39 mm SL, P<0.05) treatments and non-significantly larger than those in the 25°C treatment (mean = 32.39+0.67 mm SL, P>0.05) (Tukey HSD critical value = 2.93 for all pairwise comparisons). This may have resulted from compensatory growth [65] of the 20°C fish after the temperature treatments were terminated combined with the lower density of fish in this treatment early in the experiment (prior to culling) due to their higher mortality rates. Body shape data were size-adjusted for analysis to account for size differences among temperature treatments.

**Fig 1:**
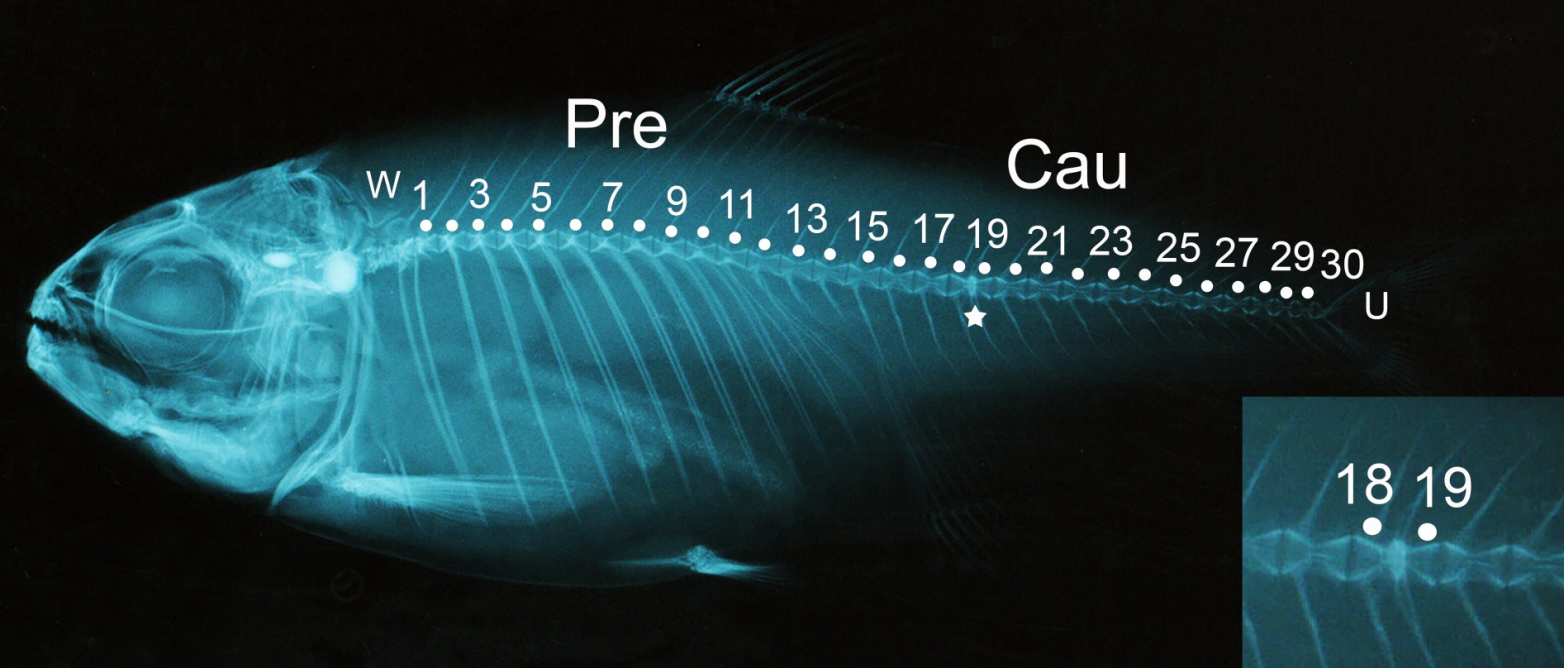
X-ray of *Astyanax mexicanus* indicating method for counting vertebrae and major anatomical structures. Vertebrae were labelled individually with points but only odd numbers are included to avoid clutter (except for the last vertebra, number 30). Four major regions were defined in the counts. 1) W: Weberian apparatus. 2) Pre: Precaudal vertebrae, include the vertebrae with ribs (1–12). 3) Cau: Caudal vertebrae include vertebrae with hemal spines (13–30). 4) U: Urostyle. The star indicates a fused vertebra that was counted as vertebrae 18 and 19. Notice the extra hemal spines and the junction formed in the middle of the vertebral body. Bottom right insert: an amplified view of the fused vertebra. The Weberian apparatus and the urostyle were not included in the counts.

Vertebral data were collected from X-rays of the specimens that were taken at the Field Museum of Natural History (Chicago, IL) using an AXR Hot Shot X-ray Machine (Associated X-ray Corporation). All specimens were X-rayed at 35 kV and 4 MA for 8 seconds. X-rays were scanned at high resolution (1,200 ppi) with an HP Scanjet G4100. Precaudal vertebrae were defined as the first vertebrae at the start of the rib cage to the last vertebrae of the end of the rib cage. Caudal vertebrae, were defined as those vertebrae lacking ribs and having hemal spines [66]. The four integrated vertebrae of the Weberian apparatus and the urostyle were not included in the vertebral counts (Fig 1). The presence of vertebral anomalies was also taken into consideration and anomalies were defined following Fraser [67] and Witten [68]. Obviously fused vertebrae were counted as separate vertebrae. Some individuals had more than one vertebral fusions or anomalies including curvature of the spine (n = 35). These individuals with “severe deformities” were included in the counts of vertebral anomalies but removed from the vertebral number and body shape analyses.

**Fig 2:**
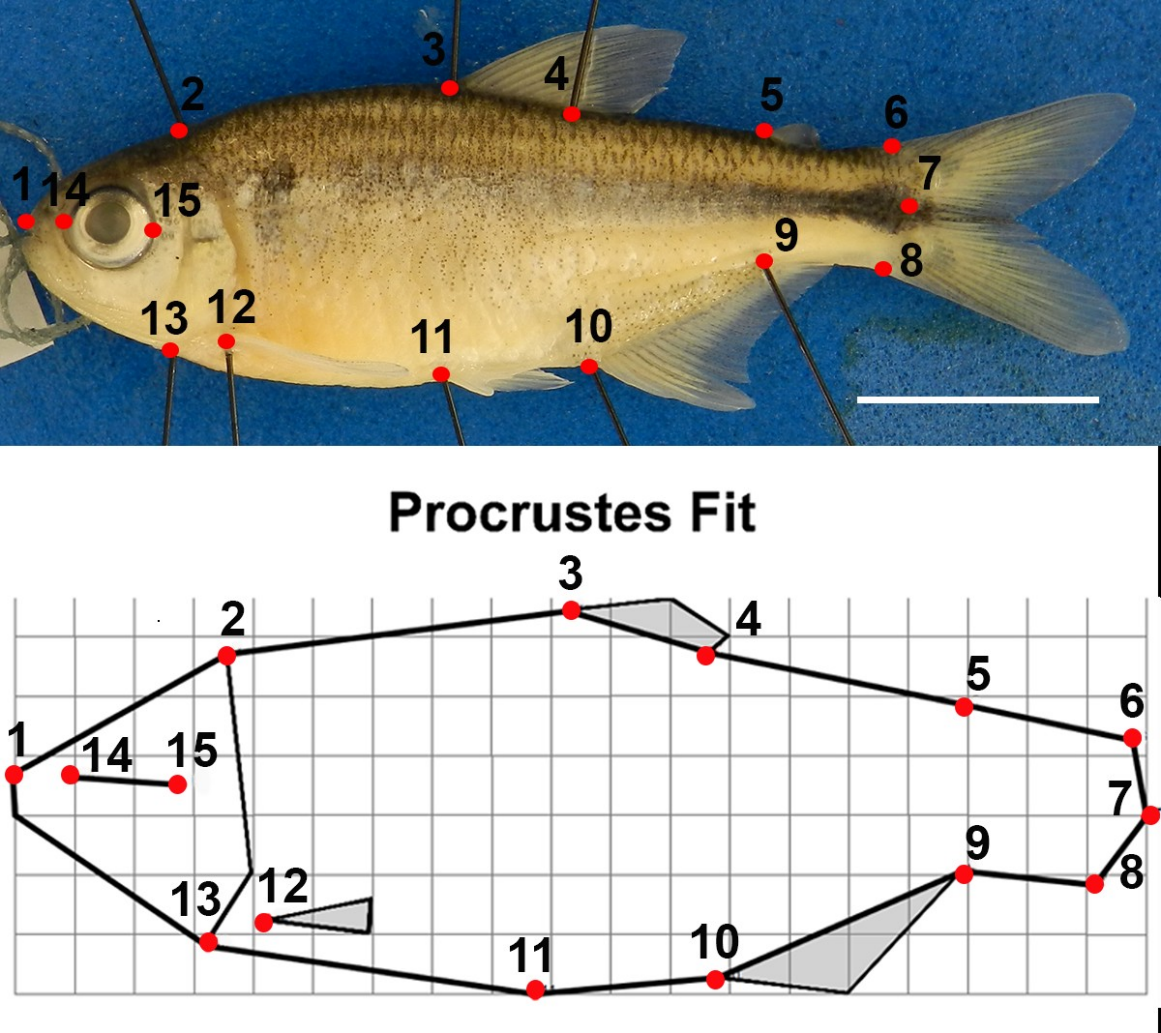
Morphometric landmarks on a specimen of *A*. *mexicanus*. Top. Photograph of a specimen indicating the position of the 15 landmarks used. 1. Most anterior point of upper jaw; 2. Supraoccipital notch immediately left-lateral to the dorsal midline (DML); 3. Anterior base of dorsal fin; 4. Posterior base of dorsal fin; 5. Anterior base of adipose fin; 6. Origin of caudal fin membrane on DML; 7. Caudal border of the hypural plate at the lateral midline; 8. Origin of caudal fin membrane on ventral midline (VML); 9. Insertion of anal fin membrane on VML; 10. Base of first anal fin ray on VML; 11. Anterior base of the pelvic fin; 12. Ventral insertion point of the left pectoral fin; 13. Most anteroventral point of coracoid; 14. Most anterior point of left eye; 15. Most posterior point of left eye. Bar indicates 10mm. Bottom. Consensus Procrustes fit of all individuals used in the study showing the mean position of each landmark. This is used as the mean body shape reference in the analysis.

Body shape data were collected from digital images of the preserved fish using geometric morphometric methods. Although preservation can have small effects on body shape [69], all fish used in this study were preserved in the same way, so preservation should not be having an effect on the differences documented among groups. Geometric morphometric analysis of body shape variation from preserved specimens is also standard practice [70–72]. Fish were straightened (if necessary) using #000 insect pins and photographed with a Nikon Coolpix P500 digital camera. Fifteen anatomical landmarks were digitized on each specimen to quantify body shape variation in two dimensions (Fig 2). To better visualize the landmarks, #000 insect pins were used to indicate the location of some of the anatomical structures in a lateral view. The landmarks were digitized using TPSDIG, version 2.17 [73], then aligned using Procrustes superimposition in MorphoJ [74]. Hereafter, this Procrustes superimposed configuration of 15 landmarks is referred to as body shape for simplicity.

### Statistical analysis

Differences in the frequency of deformities among temperature treatments were analysed using a Chi-square test of independence (Table 1). Homogeneity of variance of the vertebral counts among temperature treatments was evaluated using Levene’s test.

**Table 1:** Chi-square test of independence between temperature treatments and the number of fish with vertebral anomalies (fusions).

Temperature	20°C	23°C	25°C	28°C	Total
**No fused Vert.**	58 (75%)	65 (81%)	63 (79%)	44 (58%)	230 (73%)
**Fused Vert.**	19 (25%)	15 (19%)	17 (21%)	32 (42%)	83 (27%)
**Total**	77	80	80	76	313

The number of fish with vertebral anomalies differed significantly among temperature treatments. Relative percentages in parenthesis. (Χ^2^ = 13.23, df = 3, P = 0.0042).

We did not expect the water turbulence treatment to affect vertebral number because the turbulence treatment did not start until after vertebral number is set [60,75], but we tested for this nonetheless. A factorial ANOVA was performed in VassarStats (http://vassarstats.net/) to examine whether temperature, water turbulence, or the interaction between temperature and water turbulence significantly influenced mean tank total vertebral number, mean precaudal vertebral number, mean caudal vertebral number, or the ratio of precaudal to caudal vertebrae. We also tested the effect of water turbulence on mean tank vertebral number separately using two-sample t-tests.

To examine the effect of temperature on vertebral number in more detail, regression analyses were conducted in *R* version 3.4.0 (The R project for Statistical Computing). We fitted linear and quadratic models with tank means for each of these variables as the response variables and temperature treatment as a fixed factor. Quadratic models were included because previous studies of the effect of temperature on vertebral number indicate that the response is often non-linear (U-shaped). Linear and quadratic models were compared for each response variable using a model simplification approach. The quadratic model was only selected if the increase in the variance that it explained was statistically significant relative to the simpler linear model.

A MANCOVA was performed with centroid size (the common measure of body size used in geometric morphometrics) included as a covariate to predict the influence of temperature, water turbulence, the interaction of temperature and water turbulence, total vertebral number, and the ratio of precaudal to caudal vertebrae on the Procrustes superimposed landmarks depicting body shape variation in TPSReg, version 1.36 [76]. Wilk’s Partial η^2^ was calculated to evaluate the relative importance of each variable (Table 2). Partial η^2^ is a measure of the explanatory ability of a factor relative to unexplained variation [71].

**Table 2:** Influence of centroid size (cent. size), turbulence, temperature, total vertebral number, vertebral ratio (number of precaudal vertebrae/ number of caudal vertebrae), and temperature x turbulence (T x T), on body shape.

	Wilk's λ	Fs	df1	df2	P	Wilk's partial η^2^	Rank
**Cent. Size**	0.454	11.2	26	242	**<0.0001**	0.546	1
**Turbulence**	0.736	3.3	26	242	**<0.0001**	0.264	2
**Temp**	0.432	3.0	78	724.5	**<0.0001**	0.244	3
**Total vert.**	0.854	1.60	26	242	**0.037**	0.146	4
**Vert. Ratio**	0.859	1.53	26	242	0.052	0.141	5
**T x T**	0.659	1.39	78	724.5	**0.018**	0.130	6

Statistically significant factors are in bold. Wilk’s partial η^2^ = 1-λ^1/s^, where s = min (p, df_effect_), p = number of dependent variables in a factor, and df_effect_ = degrees of freedom of the factor of interest.

Canonical variate analysis (CVA) was conducted in MorphoJ version 1.06d [74] to examine the impact of the temperature and turbulence treatments on body shape variation. CVA is a standard multivariate method used to find the shape features that best distinguish among multiple predefined groups of specimens [77]. The analysis was conducted using the individual data without the specimens exhibiting major vertebral anomalies (n = 278) but tank averages (n = 32) were plotted to facilitate visualization. To account for the potential confounding effects of allometric variation resulting from size differences among groups, the body shape data were size-corrected. This was done by conducting a pooled within-group regression of the body shape data (response) on log_10_ centroid size (predictor) and then running the CVA on the residuals of this regression [77]. The eight treatment groups (four temperature treatments x two turbulence treatments) were used to generate the classifier variable for the CVA. Two additional CVAs with classifiers set separately by temperature treatments and turbulence treatments were also run but yielded results that were consistent with the combined CVA described above, so these are not presented. To quantify differences among groups, pairwise Mahalanobis distances were calculated between the treatment groups and permutation tests (10,000 permutations) were used to test for significance in MorphoJ. CVA scores were exported to TPSRegr v. 1.45 to generate the deformation grids showing how body shape diverges along the CV axes.

## Results

Temperature had significant effects on all morphological traits examined including the frequency of vertebral anomalies, variation in vertebral counts, the mean number of vertebrae, the ratio of precaudal to caudal vertebrae, and body shape. Water turbulence also impacted body shape in the manner predicted but did not significantly impact any of the vertebral variables measured.

### Vertebrae are affected by developmental temperature

Total vertebral number ranged from 26 to 31 across all treatments and resulted from 11 different combinations of precaudal and caudal vertebral counts (S1 Table). The number of precaudal vertebrae ranged from 11 to 13, while caudal vertebral counts were more variable and ranged from 14 to 19. Vertebral anomalies (Fig 1) differed significantly between groups with the extreme 20°C and especially the 28°C temperature treatments showing higher percentages of individuals with vertebral anomalies than the intermediate temperature treatments, 23°C and 25°C (Table 1). Variance in vertebral number also differed among temperature treatments for total vertebral number (Levene’s test, W = 6.16, df = 3, 309, P<0.001) and precaudal vertebral number (W = 10.66, df = 3, 309, P<0.001) but not for caudal vertebral number (W = 0.463, df = 3, 309, P = 0.709). Variance in precaudal vertebral number followed the pattern seen with the prevalence of vertebral fusions, greater variance in the extreme temperature treatments. However, there was no obvious pattern for differences in variance with temperature for total vertebral number.

As expected given that the water turbulence treatment started after vertebral number is set, there was no association between the water turbulence treatment and any of the vertebral traits measured either when tested in conjunction with temperature (Factorial ANOVA, F≤0.28, P≥0.602, DF = 1, 31 for all vertebral traits; the interaction was not significant either) or separately (two-sample t-test, t≤0.47, DF = 30, P≥0.642 for all vertebral traits). The mean number of total vertebrae, precaudal vertebrae, caudal vertebrae, and the ratio of precaudal to caudal vertebrae were all very similar between the no added water turbulence and the added water turbulence treatments (29.699 vs. 29.681, 12.064 vs. 12.096, 17.636 vs. 17.586, and 0.686 vs. 0.689 respectively).

**Fig 3:**
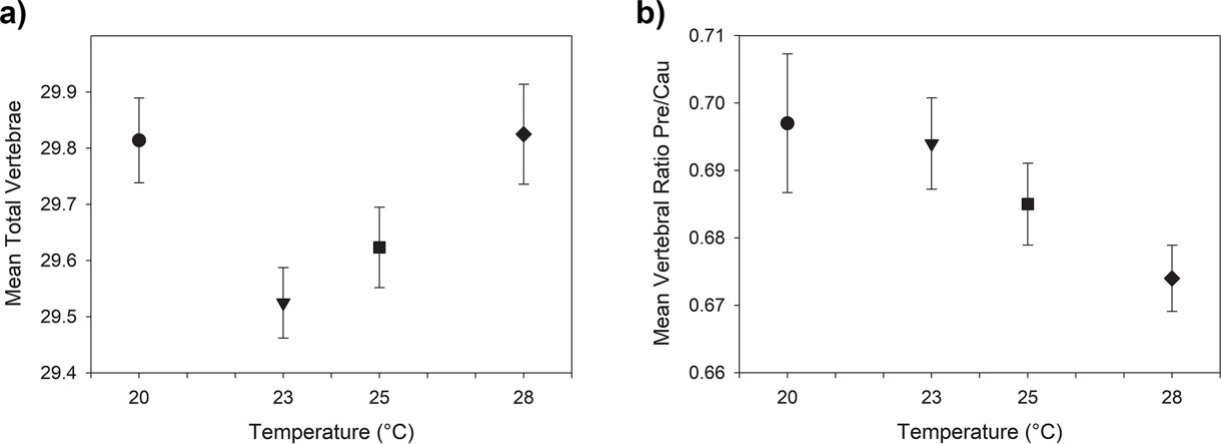
Summary of vertebral counts. Tank means of total number of vertebrae and vertebral ratio per temperature treatments. a) Mean total number of vertebrae. b) Vertebral ratio defined by the number of Precaudal and Caudal vertebrae (Pre/Cau). Error bars: Standard error of the mean.

The mean total number of vertebrae per tank exhibited a U-shaped distribution across the temperature treatments. The lowest and highest temperature treatments (20°C and 28°C) resulted in similarly high means relative to the intermediate temperatures (Fig 3A and S2 Table). The relationship between temperature treatment and mean tank total vertebral number was statistically significant when a quadratic model was fit (r^2^ = 0.268, P = 0.003). Temperature was also significantly associated with the number of precaudal vertebrae, which decreased with temperature when a linear model was fit (r^2^ = 0.216, P = 0.007), but not with caudal vertebrae with either linear or quadratic models (r^2^ = 0.093, P = 0.089). The ratio of precaudal to caudal vertebrae exhibited a declining linear trend with temperature that was statistically significant when a linear model was fitted (r^2^ = 0.165, P = 0.021). Fish in the 20°C treatment have relatively more precaudal vertebrae and fish in the 28°C treatment have relatively more caudal vertebrae despite having similar mean total vertebral counts (Fig 3B).

### Water turbulence and temperature affect body shape

**Fig 4:**
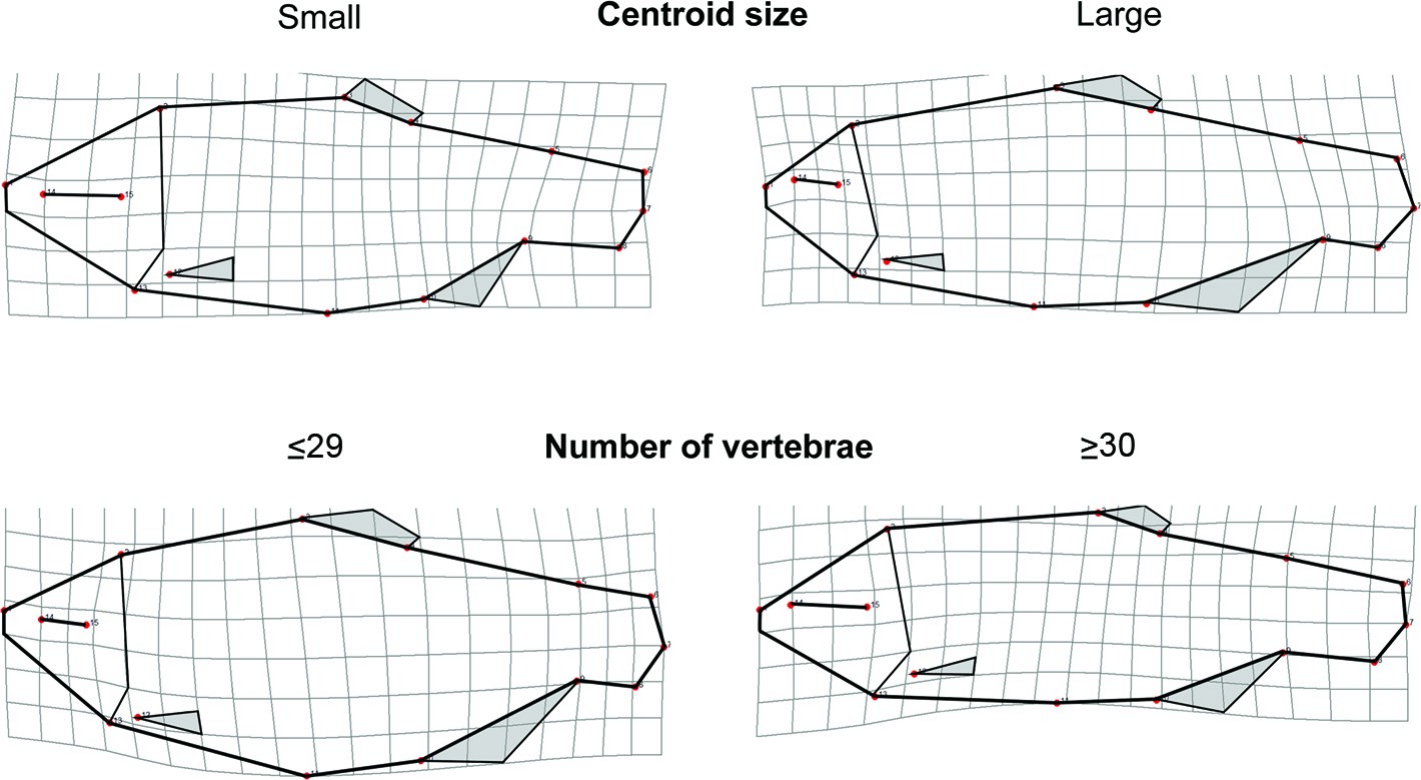
Body shape change associated with centroid size and vertebral number. Top left: estimated shape of the smallest individuals in the study (based on predicted shape of the smallest individual from a regression of body shape on body size, with body size estimated using centroid size). Top right: Estimated shape of the largest individuals in the study. Bottom left: Estimated shape of an individual with fewer total vertebrae (≤29). Bottom right: Estimated shape of an individual with more total vertebrae (≥30). Specimens were divided into these vertebral classes based on the frequencies of total vertebral number across treatments. Specimens with less than 29 or more than 30 vertebrae were uncommon. The deformation grids were created in TPSRegr v. 1.45 by regressing body shape on centroid size and vertebral number, respectively. Shape differences are exaggerated by a factor of 3 to accentuate the shape changes and facilitate their visualization.

The MANCOVA analysis indicated that most factors in the model significantly influenced body shape (Table 2). Wilk’s partial η^2^ ranked centroid size (a measure of body size) as the factor that explained the greatest amount of variation in body shape, accounting for approximately 55% of the partial variance. Body shape variation attributable to differences in size (allometry) was associated largely with the head and tail regions and with body depth (Fig 4). Turbulence and temperature were the next most important variables accounting for similar percentages of partial variance, 26.4 and 24.4%, respectively. Body shape variation associated with these variables is described below (CVA section). The other variables accounted for less variation in body shape and were of marginal statistical significance (or marginally insignificant): total vertebral number (14.6%), vertebral ratio (14.1%), and the interaction between turbulence and temperature (13.0%). Although vertebral number variation did not account for a large amount of the variation in body shape and was only marginally significant, the variation observed was consistent with our prediction. Fish with fewer vertebrae [26–29] tended to have deeper bodies, and smaller heads and eyes than fish with more vertebrae [30–31], even when allometry, temperature and turbulence treatments were accounted for (Fig 4).

**Fig 5:**
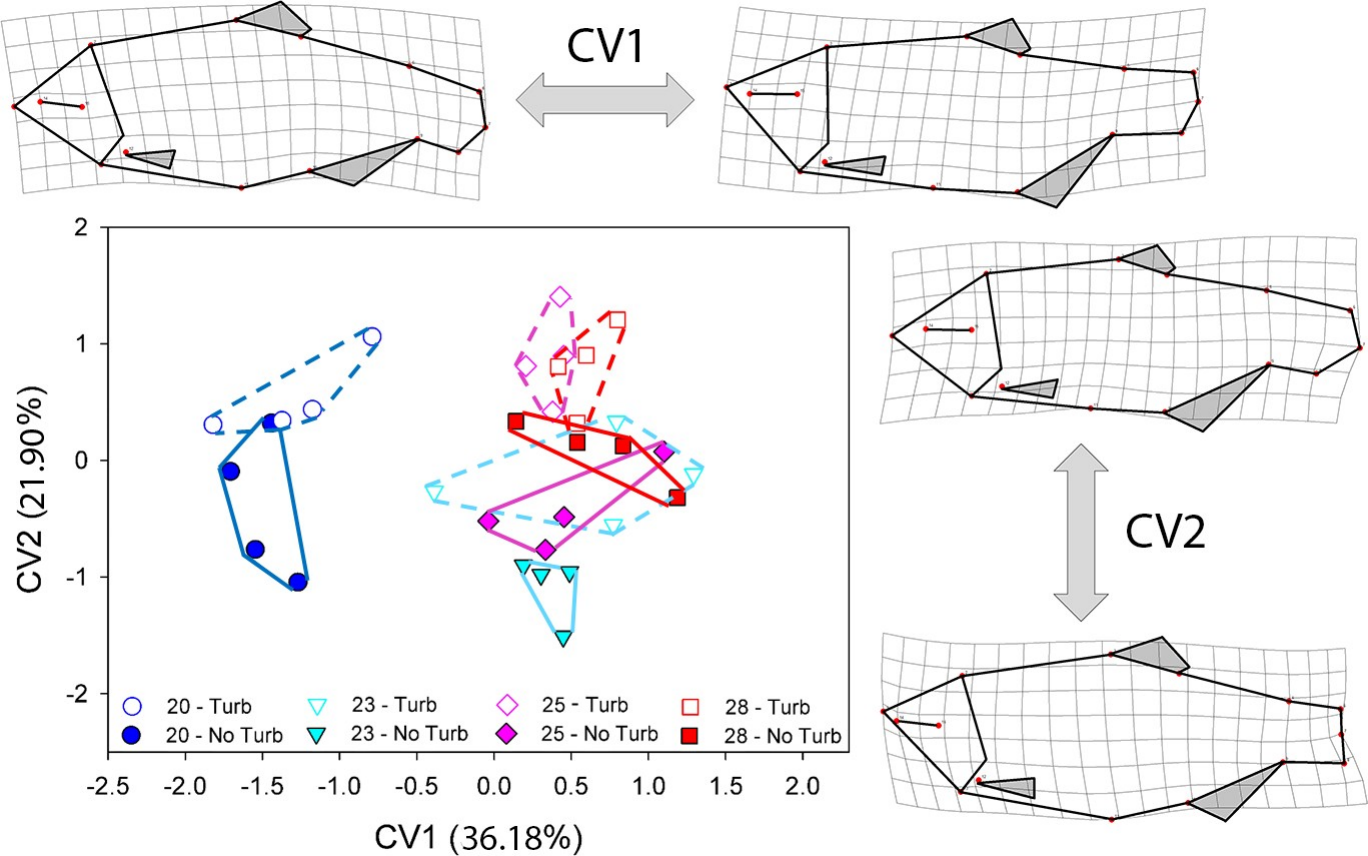
Canonical variate analysis (CVA) and predicted shapes based on body shape variation by treatments. The CVA shows separation between groups (temperature and water turbulence treatments) based on the tank means. Lines are convex hull around the group means. The deformation grids were created in TPSRegr v. 1.45 by regressing body shape on the CV scores. Shape differences are exaggerated by a factor of 3 to accentuate the shape changes and facilitate their visualization.

The CVA of the body shape data resulted in good separation among tank means based on the treatment groups (combination of temperature and turbulence treatments) and indicated that body shape differed significantly among most groups. Body shape variation attributable to CV1 was associated primarily with differences in the head, fin positions and fin base lengths, and the caudal peduncle, with fish from the 20°C treatment differing from fish in the other temperature treatments along this axis (Fig 5). CV2 was primarily associated with divergence in body shape related to the turbulence treatments. Fish subjected to the added turbulence treatment always varied towards the more positive end of CV2 and had more streamlined bodies relative to those subjected to the no added turbulence treatment.

All but two pairwise Mahalanobis distances (M) among treatment groups were significant, with the non-significant distances being between similar treatments (S3 Table). The magnitude of the pairwise distances was greatest between groups at the temperature extremes.

## Discussion

This study highlights how differences in temperature can have notable impacts on adaptively important morphological traits and that different traits respond in different ways to temperature. Differences in water turbulence were also associated with divergence in body shape in a predictable direction, although the interaction between temperature and water turbulence had only a small effect on body shape.

### Temperature effects on vertebrae

The increase in variance in vertebral fusions at the more extreme temperature treatments may be explained by developmental instability and the breakdown of canalization mechanisms that reduce variation around the optimum phenotype [9,21,78,79]. Vertebral fusions were particularly common in the highest temperature treatment, indicating that this temperature had a particularly large impact on the development of the vertebral column. However, the baseline rate of vertebral anomalies was still relatively high (19–21% for the 23°C and 25°C treatments, respectively), which may be a consequence of developing in a lab setting since the rate of vertebral fusions and anomalies is unlikely to be this high in the wild. Individual responses to variation in environmental conditions will often translate to higher phenotypic variation [21,79], which could accelerate the population’s response to selective pressures [7]. Future studies on the impact of vertebral anomalies on body shape and swimming performance in this species would likely be quite interesting given the frequency with which they arise in a lab setting. Interestingly, increases in variance at temperature extremes in other vertebral traits and body shape were not as apparent, suggesting trait specific robustness to environmental variation during development.

The U-shaped relationship that we found between temperature treatment and total vertebral number is a relatively common response for intraspecific variation under different temperature treatments in fishes [33], although it is not universal [16]. Interestingly, despite having very similar numbers of total vertebrae, fish at the temperature extremes differed in the ratio of precaudal to caudal vertebrae. There were more precaudal vertebrae in individuals reared at 20°C and more caudal vertebrae at 28°C. Precaudal vertebrae support ribs, which serve as attachment sites for swimming muscles, and are in the region of the body housing the viscera, while the caudal vertebrae provide support for the muscular tail, which is particularly important for swimming performance. Thus, differences in the relative numbers of precaudal and caudal vertebrae may reflect differences in swimming performance. For example, Swain’s [37] classic study found significant differences in C-start reactions in young threespine stickleback differing in the ratio of precaudal to caudal vertebrae but not in total vertebral number (although the optimal ratio changed with size). It is unclear whether the magnitude of the difference seen here, which was approximately 9.84% of the range of variation seen among individuals, is functionally significant. Nonetheless, it raises the interesting possibility that increases and decreases in temperature may affect swimming performance of *A*. *mexicanus* in different ways, despite similarities in total vertebral number.

There was a greater range of variation in the number of caudal vertebrae [14–19] than in the number of precaudal vertebrae [12–14], although it was the number of precaudal vertebrae that differed significantly among temperature treatments. Greater variation in the number of caudal vertebrae has been documented frequently in fishes [16,27,80,81]. Interestingly, Dowling et al. [82] reported differences in the number of precaudal vertebrae associated with body shape differences between two cavefish clades of *A*. *mexicanus* based on a phylogeographic analysis of mitochondrial DNA. They found that clade A cavefish had the ancestral number of 12 precaudal vertebrae (which was also the most common number of precaudal vertebrae observed in our study), whereas clade B cavefish had 11 precaudal vertebrae, which was associated with a compression of the body along the anterior-posterior axis. Thus, precaudal vertebral number seems prone to diverge in *A*. *mexicanus* both among naturally occurring populations and in lab strains in response to variation in developmental temperature.

Although the general patterns shown in this study are clear, the processes through which temperature results in changes in the number and identity of vertebrae are not known. One possibility could be alterations in the timing of somite formation, perhaps affecting the cell cycle, may be at play since the number of vertebrae is related to the number of somites that form during embryogenesis [22,61,83]. There is a somatic clock involved in their development resulting in somites forming at specific time intervals as the body axis extends posteriorly until the presomitic mesoderm in the tailbud is exhausted [84,85]. Somite number may change if the rate of individual somite formation changes relative to the rate of growth of the body axis or if the body axis growth period is altered [25,26]. Ward and Brainerd [25] also suggest that shifts in *Hox* gene expression boundaries may be associated with changes in the ratio of precaudal to caudal vertebrae and that longer periods of somitogenesis in the tail may be associated with an increase in the number of caudal vertebrae. Understanding the molecular and cellular details of how temperature results in changes in vertebral number is a major direction for future research.

### Water turbulence and temperature affect body shape

Temperature also significantly impacted body shape. The strongest effect was on the fish in the 20°C treatment relative to the other treatments. In our model, temperature ranked behind allometry but was very close to the water turbulence treatment as a factor contributing to body shape variation (Table 2). It is important to note that temperature could have had a greater effect on body shape if the fish were maintained in the temperature treatments for the whole experiment. By ending the temperature treatment after one month, this study focused on the effects of temperature differences during early development only. Our finding of significant differences in body shape related to temperature, even when limiting the treatment only to the first month of development, highlights the importance of temperature on the phenotypes of fishes.

The effect of temperature on body shape variation has been reported previously in fishes [39,86,87]. Some of the components of body shape that varied with temperature in our study were similar to those described in other studies in fishes indicating that changes in the positioning of the paired fins and head size may be common responses to temperature during development [39,86,87]. Why temperature influences body shape is not clear. A component of this may be related to the effect of temperature on somite and vertebral number, which may impact the body length/depth relationship. Greater body depth of fish reared at warmer temperatures appears to be a relatively common response [39,86]. However, temperature is also known to impact growth rate, food intake, and food conversion efficiency [88], which may influence how body shape develops as fish grow. That is, differences in environmental temperature may alter the ability of fish to process food and incorporate it into their tissue, resulting in small but significant differences in body shape when comparing fish of similar sizes, including possibly differences in body depth.

Locomotion abilities are presumably under strong selection pressures since all activities of fish are highly dependent on effective movement and aquatic environments are energetically demanding [46,89,90]. As predicted, individuals subjected to additional turbulence had more streamlined bodies compared to individuals that were reared in standing water, which is consistent with expectations for body shape divergence of populations adapting to habitats with greater water movement (Fig 5). A more streamlined body in fishes reduces drag, thus reducing the energy requirements for the individuals to maintain their position when swimming against strong currents [49]. The results are also consistent with the difference in body depth seen between the surface form of *A*. *mexicanus*, which inhabits faster moving rivers and streams and is more streamlined, and the cave form, which inhabits standing waters in cave pools and has a deeper body [64]. The effect of water turbulence on body shape would be an effect that is additional to the shape variation attributable to that of the temperature treatments described above. Thus, body shape is influenced by several different factors acting together during development in complex ways.

### Vertebral number—body shape covariation and its potential functional implications

Although the relationship between vertebral number and body shape was not strong, it was statistically significant and in the direction predicted: fish with more vertebrae had more streamlined bodies. Across broad taxonomic ranks, vertebral number and body shape are obviously associated in vertebrates. Increases in body elongation are strongly correlated with increases in vertebral number [36,91]. Although at microevolutionary scales, difference in vertebral length or factors impacting growth along the body depth axis independent of the body length axis may also have a strong influence, associations between body shape and vertebral number are increasingly being reported [17,27,29,92]. Moreover, there often appears to be body region specificity in the pattern of vertebral-body form covariation. As indicated above, variation in the ratio of precaudal to caudal vertebrae can significantly affect swimming performance [16,37]. If vertebral number and body shape are integrated and generally covary in fishes, then studies of swimming performance should take both into account. Although we do not have evidence that the phenotypic variation documented here for *A*. *mexicanus* affects swimming performance, this is something worthy of future study given the potential fitness implications.

### Broader implications

Beyond providing greater insight into the sources of phenotypic variation observed in nature, studying phenotypic plasticity in more detail may provide insight into how and when new traits arise as developmental pathways are modified by changing environmental conditions [7,93]. In this study, different vertebral traits responded in different ways to temperature and different aspects of body shape were influenced by variation in temperature and water turbulence. The variety of plastic responses result in sets of individual phenotypes that likely vary in fitness. The wide range of plastic responses may thus include some precursors to adaptive evolution and it is interesting to reflect on how standing patterns of phenotypic plasticity may influence the evolutionary trajectory of lineages [7,11,93]. In a broader context, our results offer another example of how global climate change could directly impact adaptively important traits [94–96].

## Supporting information

S1 Table Vertebral phenotype by temperature treatments. (DOCX)

S2 Table Average body size and vertebral data by tank. (DOCX)

S3 Table Pairwise mahalanobis distances between treatment groups. (DOCX)

S1 Appendix Water velocity estimation. (DOCX)
