# Supplementary material for: Effects of temperature and water turbulence on vertebral number and body shape in Astyanax mexicanus (Teleostei: Characidae)
Source: PLoS One. 2019 Jul 29;14(7):e0219677. doi: 10.1371/journal.pone.0219677 (PMC6663064; doi:10.1371/journal.pone.0219677)
Supplement: S1 Table — (DOCX) [file pone.0219677.s001.docx]

**Table S1.** Vertebral phenotypes by temperature treatment. Tot V is total vertebral number and Pre/Cd is the number of precaudal/caudal vertebrae. Specimens with severe vertebral anomalies are not included.

**Tot V 26 28 29 30 31 Total**

**Pre/Cd 12/14 11/17 12/16 11/18 12/17 13/16 11/19 12/18 13/17 12/19 13/18**

**Temp 20** 0 2 1 0 8 4 2 36 14 3 2 72

**23** 0 0 0 1 32 6 1 30 2 3 0 75

**25** 1 1 1 2 22 2 0 34 1 6 1 71

**28** 0 2 1 3 12 1 1 28 3 8 1 60

**Total**  1 5 3 6 74 13 4 128 20 20 4 278
