# Supplementary material for: Effects of temperature and water turbulence on vertebral number and body shape in Astyanax mexicanus (Teleostei: Characidae)
Source: PLoS One. 2019 Jul 29;14(7):e0219677. doi: 10.1371/journal.pone.0219677 (PMC6663064; doi:10.1371/journal.pone.0219677)
Supplement: S2 Table — (DOCX) [file pone.0219677.s002.docx]

**Table S2.** Average body size and vertebral data by tank. N is the number of specimens in each tank, N* is the number of specimens after specimens with severe vertebral anomalies were removed, Temp is the temperature treatment in ºC, Flow is the water turbulence treatment with 0 being the no additional turbulence treatment and 1 being the additional turbulence treatment. SL is the standard length measured in mm, Csz is the centroid size, Pre is the average number of precaudal vertebrae, Cd is the average number of caudal vertebrae, Tot_V is the average number of total vertebrae, and Pre/Cd is the ratio of precaudal vertebrae to caudal vertebrae. Tank averages are those calculated after the specimens with severe vertebral anomalies were removed.

**Tank N N* Temp Flow SL Csz Pre Cd Tot_V Pre/Cd**

2 10 9 20 0 32.03 47.97 12.00 17.78 29.78 0.676

7 9 9 20 0 32.57 49.07 12.00 17.89 29.89 0.672

11 10 10 20 0 33.67 51.20 12.20 17.90 30.10 0.684

20 10 9 20 0 34.64 53.33 12.56 17.11 29.67 0.736

3 10 9 20 1 35.78 53.43 12.00 17.67 29.67 0.679

6 9 9 20 1 34.35 52.91 12.22 17.89 30.11 0.685

8 10 9 20 1 35.23 54.53 12.22 17.56 29.78 0.698

19 9 8 20 1 38.84 58.45 12.63 16.88 29.50 0.749

4 10 8 23 0 34.42 50.36 12.25 17.38 29.63 0.709

16 10 10 23 0 28.35 44.76 12.20 17.10 29.30 0.715

17 10 10 23 0 27.70 41.95 12.10 17.50 29.60 0.692

26 10 10 23 0 32.35 50.29 11.80 17.80 29.60 0.664

12 10 10 23 1 32.47 50.52 12.30 17.20 29.50 0.718

30 10 9 23 1 30.16 45.55 12.00 17.22 29.22 0.697

31 10 8 23 1 31.23 48.07 12.00 17.75 29.75 0.676

34 10 10 23 1 31.07 47.76 12.00 17.60 29.60 0.683

21 10 9 25 0 35.05 52.56 12.00 17.67 29.67 0.680

23 10 9 25 0 32.31 49.18 12.00 17.33 29.33 0.697

25 10 10 25 0 32.43 51.31 12.00 17.90 29.90 0.671

32 10 7 25 0 30.89 49.76 12.00 17.71 29.71 0.678

5 10 8 25 1 32.74 47.55 12.38 17.25 29.63 0.721

15 10 10 25 1 32.56 48.71 12.00 17.60 29.60 0.683

18 10 9 25 1 31.39 47.00 11.89 17.89 29.78 0.665

33 10 9 25 1 31.80 48.02 11.89 17.44 29.33 0.682

1 10 9 28 0 33.23 48.06 11.78 17.78 29.56 0.663

14 10 8 28 0 27.55 40.62 12.13 17.75 29.88 0.686

22 7 5 28 0 34.99 53.87 12.00 18.00 30.00 0.667

28 10 7 28 0 29.37 46.93 12.00 17.57 29.57 0.683

9 10 8 28 1 31.09 46.23 11.88 18.25 30.13 0.652

27 10 6 28 1 28.45 48.83 12.00 18.00 30.00 0.668

35 9 8 28 1 33.65 51.68 12.13 17.50 29.63 0.694

36 10 9 28 1 31.30 46.82 12.00 17.67 29.67 0.681

**Total= 313 278**
