# Supplementary material for: Effects of temperature and water turbulence on vertebral number and body shape in Astyanax mexicanus (Teleostei: Characidae)
Source: PLoS One. 2019 Jul 29;14(7):e0219677. doi: 10.1371/journal.pone.0219677 (PMC6663064; doi:10.1371/journal.pone.0219677)
Supplement: S3 Table — (DOCX) [file pone.0219677.s003.docx]

**Table S3. Pairwise Mahalanobis distances between treatment groups**. Mahalanobis distances are below the diagonal, probability values from permutation tests (10,000 permutation rounds) are above the diagonal. Numbers in top row and first column indicate the temperature treatments (20°C, 23°C, 25°C, and 28°C) and letters indicate the turbulence (T) or no turbulence (NT) treatments. With two exceptions (23°C-NT vs. 25°C-NT and 25°C-T vs. 28°C-T), pairwise Mahalanobis distances between treatment groups were significant.

**20°C-NT 20°C-T 23°C-NT 23°C-T 25°C-NT 25°C-T 28°C-NT 28°C-T**

**20°C-NT** - 0.001 <0.001 <0.001 <0.001 <0.001 <0.001 <0.001

**20°C-T** 1.755 - <0.001 <0.001 <0.001 <0.001 <0.001 <0.001

**23°C-NT** 2.380 2.415 - <0.001 0.325 <0.001 <0.001 <0.001

**23°C-T** 2.489 2.475 1.848 - <0.001 <0.001 <0.001 <0.001

**25°C-NT** 2.198 2.259 1.225 1.694 - <0.001 0.001 0.010

**25°C-T** 2.449 2.059 2.167 1.708 1.667 - <0.001 0.333

**28°C-NT** 2.600 2.675 2.204 2.151 1.834 2.015 - 0.002

**28°C-T** 2.624 2.234 2.112 1.928 1.592 1.293 1.967 -
