# Supplementary material for: Effects of temperature and water turbulence on vertebral number and body shape in Astyanax mexicanus (Teleostei: Characidae)
Source: PLoS One. 2019 Jul 29;14(7):e0219677. doi: 10.1371/journal.pone.0219677 (PMC6663064; doi:10.1371/journal.pone.0219677)
Supplement: S1 Appendix — (DOCX) [file pone.0219677.s004.docx]

**APPENDIX 1**

**Water Velocity Estimation:**

To estimate the velocity of water movement in the water turbulence treatments, a positively buoyant object was placed close to the outlet tube of the water pump and released, and the time it took to get to the other side of the tank was measured. This was repeated three times per water pump and an average water velocity was calculated. This represented the measure of maximum water velocity. To assess what water velocity was like in other parts of the tank, a dissolution-based method was employed. Jokiel and Morrissey (1) found that the rate of dissolution of solids is linearly related to the velocity of water flow until the surface area falls below about 30%. Linearity was assumed. During the experiment, pieces of sucrose candy (Lifesavers™) were placed throughout the tank and their weight before and after a set time in the water under the flow treatment was measured following the approach generally outlined by Koehl and Alberte (2). The percentage of weight loss was calculated based on the differences in weight. In total, 27 pieces of candy were placed at approximately equal distance throughout the tank. These were distributed in three sets of nine. Each set of three by three candies was suspended using zip ties; each zip tie was separated by 5cm (left, center and right), and the candies were placed at distances of 7 cm from one another along the zip tie (surface, middle and bottom). The three sets were separated by 9 cm from each other. The experiment was run for two minutes and the candy were removed and left to air dry for 24 hours before the weight was recorded. A control without the pumps with the same set up was also used. This experiment was run twice with water pumps picked at random. The maximum average velocity was 0.26 m s^-1^ in the surface center of the tank in front of the pump based on the previous experimental run. The surface left side was estimated to run about 0.16 m s^-1^, and the right side at 0.21 m s^-1^. This difference may be due to the design of the pump outlet, which is tilted slightly to the right. The middle depth velocity was estimated to be 0.17 m s^-1^ in the center of the tank, 0.19 on the left side, and 0.17 m s^-1^ on the right. At the bottom of the tank, the estimated velocities were 0.19 m s^-1^ at the center, 0.19 m s^-1^ on the left side, and 0.18 m s^-1^ on the right.

**References:**

1. Jokiel PL, Morrissey JI. Water motion on coral reefs: evaluation of the’clod card’ technique. Mar Ecol Prog Ser. 1993;175–81.

2. Koehl MAR, Alberte RS. Flow, flapping, and photosynthesis ofNereocystis leutkeana: a functional comparison of undulate and flat blade morphologies. Mar Biol. 1988;99(3):435–44.
